# Changes in the Glucose Concentration Affect the Formation of Humic-like Substances in Polyphenol–Maillard Reactions Involving Gibbsite

**DOI:** 10.3390/molecules29092115

**Published:** 2024-05-03

**Authors:** Nan Wang, Yongquan Cui, Yanhui Zhou, Pingxin Liu, Mingshuo Wang, Haihang Sun, Yubao Huang, Shuai Wang

**Affiliations:** 1 College of Agriculture, Jilin Agricultural Science and Technology University, Jilin 132101, China; wangnan664806@126.com (N.W.); 15043962779@163.com (Y.C.); liupingxin2023@126.com (P.L.); 13684766706@163.com (M.W.); 18843840365@163.com (H.S.); hyb885329@163.com (Y.H.); 2 Agricultural Technology Extension Station of Jiaohe City, Jiaohe 132500, China; jhzyh0815@163.com

**Keywords:** polyphenol–Maillard reaction, humic-like substance, glucose, abiotic condensation, gibbsite

## Abstract

The polyphenol–Maillard reaction is considered one of the important pathways in the formation of humic-like substances (HLSs). Glucose serves as a microbial energy source that drives the humification process. However, the effects of changes in glucose, particularly its concentration, on abiotic pathways remain unclear. Given that the polyphenol–Maillard reaction requires high precursor concentrations and elevated temperatures (which are not present in soil), gibbsite was used as a catalyst to overcome energetic barriers. Catechol and glycine were introduced in fixed concentrations into a phosphate-buffered solution containing gibbsite using the liquid shake-flask incubation method, while the concentration of glucose was controlled in a sterile incubation system. The supernatant fluid and HLS components were dynamically extracted over a period of 360 h for analysis, thus revealing the influence of different glucose concentrations on abiotic humification pathways. The results showed the following: (1) The addition of glucose led to a higher degree of aromatic condensation in the supernatant fluid. In contrast, the supernatant fluid without glucose (Glu0) and the control group without any Maillard precursor (CK control group) exhibited lower degrees of aromatic condensation. Although the total organic C (TOC) content in the supernatant fluid decreased in all treatments during the incubation period, the addition of Maillard precursors effectively mitigated the decreasing trend of TOC content. (2) While the C content of humic-like acid (C_HLA_) and the C_HLA_/C_FLA_ ratio (the ratio of humic-like acid to fulvic-like acid) showed varying increases after incubation, the addition of Maillard precursors resulted in a more noticeable increase in C_HLA_ content and the C_HLA_/C_FLA_ ratio compared to the CK control group. This indicated that more FLA was converted into HLA, which exhibited a higher degree of condensation and humification, thus improving the quality of HLS. The addition of glycine and catechol without glucose or with a glucose concentration of 0.06 mol/L was particularly beneficial in enhancing the degree of HLA humification. Furthermore, the presence of glycine and catechol, as well as higher concentrations of glucose, promoted the production of N-containing compounds in HLA. (3) The presence of Maillard precursors enhanced the stretching vibration of the hydroxyl group (–OH) of HLA. After the polyphenol–Maillard reaction of glycine and catechol with glucose concentrations of 0, 0.03, 0.06, 0.12, or 0.24 mol/L, the aromatic C structure in HLA products increased, while the carboxyl group decreased. The presence of Maillard precursors facilitated the accumulation of polysaccharides in HLA with higher glucose concentrations, ultimately promoting the formation of Al–O bonds. However, the quantities of phenolic groups and phenols in HLA decreased to varying extents.

## 1. Introduction

The formation of humic substances (HSs) has long been a topic of interest for scholars in soil and environmental science. Previous studies have primarily focused on biotic pathways, with limited attention given to abiotic pathways [1]. Under abiotic conditions, substances similar to HSs can be formed through a condensation of various precursors, yet they lack the complexity of genuine HSs found in nature, and are hence referred to as humic-like substances (HLSs). The formation of HLSs through abiotic pathways can be elucidated using the phenol–protein and Maillard theories [2]. The polyphenol–Maillard reaction, a nonenzymic browning process involving the polycondensation of sugars and amino acids, is particularly noteworthy as an abiotic pathway leading to the formation of HLSs [3]. This reaction can occur spontaneously when heat is applied without the involvement of microorganisms or biological enzymes. During this process, proteins and sugars undergo cleavage, rearrangement, and polymerization, resulting in the formation of N-containing heterocycles [4], which are characteristic of both HSs and HLSs. In the formation process of HSs or HLSs, biomacromolecules such as proteins, polysaccharides, lignins, and polyphenols are broken down into smaller molecules, including amino acids, sugars, and quinones. These smaller molecules, known as humic precursors, then undergo polycondensation reactions to form dark-colored polymers resembling HLSs [5]. It is widely recognized that the recombination of humic precursors is one of the pathways involved in the formation of HSs or HLSs.

Glucose, as a humic precursor, plays a crucial role in the formation and nature of HSs, while also affecting abiotic pathways of humification and associated products in natural environments [6]. Recent research has highlighted the importance of reducing sugars and polysaccharides, derived from the degradation of lignocellulose, as essential precursors for the formation of humus materials. These precursors can combine with amino acids to synthesize HSs [7]. The addition of exogenous precursors has been found to enhance the interconnection among various precursors and further facilitate the humification process [8]. Several studies have shown that the concentration of sugars has a substantial influence on the degree of humification [4]. Polysaccharides, as primary energy and C sources, are considered major contributors to the formation of HSs, particularly in the polyphenol–Maillard reaction, where they can facilitate the conversion of fulvic acid (FA) to humic acid (HA) [9]. Chen et al. [10] observed that the supplementation of sugars could enhance the humification process and modify the content of HS precursors, thereby enhancing the quality of chicken manure compost for C sequestration. In his report, Bui et al. [11] highlighted the polyphenol–Maillard reaction proposed by Maillard in 1913, which involved a condensation reaction between reducing sugars and amino acids. In the presence of catalysts, sugars could undergo auto-oxidation to produce dicarbonyl compounds, which play a key role in the formation of melanoidins.

Reducing sugars, such as glucose, play a vital role in driving the humification process by serving as an energy source for microorganisms. However, the contribution of reducing sugars to HLS formation via the Maillard pathway in abiotic processes is unclear. Previous research has mainly focused on the abiotic formation of HSs by polyphenols [12], while studies on the abiotic formation of HSs by glucose have been limited [13]. In nature, metallic oxides coexist with organic matter and have been found to have varying degrees of influence on humification [14]. Si, Mn, Al, and Fe oxides have been shown to aid in the breakdown of reaction mixtures containing humic precursors [5]. Under aerobic conditions, the presence of oxides containing Mn, Al, Fe, and Si has been found to enhance the condensation of precursors and increase the HLA yield and degree of humification [15]. However, limited literature exists on the use of gibbsite as a catalyst for investigating the polyphenol–Maillard reaction. Therefore, the goal of this research was to examine the impact of differing concentrations of glucose on the polyphenol–Maillard reaction and understand the role of glucose in promoting abiotic humification based on the characteristics of related products under abiotic stress. To address this gap, the liquid shake-flask incubation method was employed. This involved adding catechol and glycine solutions to a phosphate buffer containing gibbsite, while only controlling the glucose concentration in the sterile incubation system. Gibbsite was used as a catalyst to overcome energetic barriers due to the high precursor concentrations and elevated temperatures required for the polyphenol–Maillard reaction. The supernatant fluid was collected over time to measure the *E*_4_/*E*_6_ ratio, TOC content, and humus composition. Furthermore, the FTIR spectra and elemental composition of the extracted HLA from the remaining incubation medium were analyzed. The resulting findings helped to elucidate the effect of glucose on abiotic humification catalyzed by gibbsite, contributing to our understanding of the mechanism of abiotic humification and the pathways of humus formation.

## 2. Results

### 2.1. E_4_/E_6_ Ratio and Total Organic C (TOC) of the Supernatant Fluid

**Figure 1:**
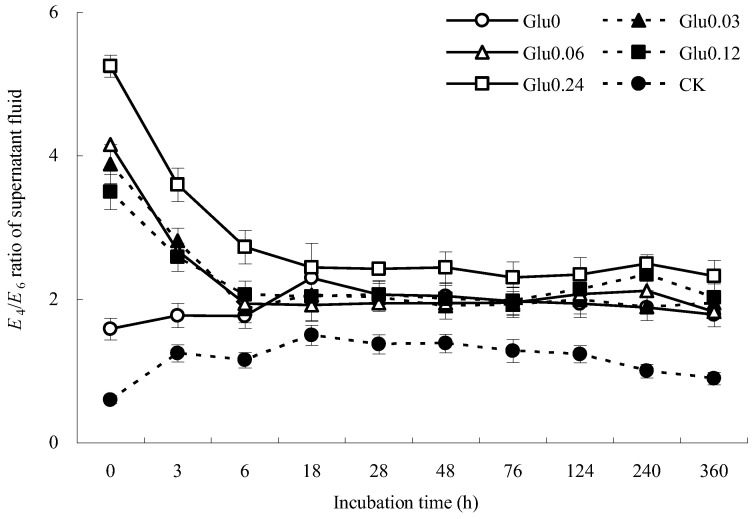
Impact of glucose concentration variations on the *E*_4_/*E*_6_ ratio of the supernatant fluid. Note: Different glucose concentrations (0, 0.03, 0.06, 0.12, and 0.24 mol/L) were labeled as Glu0, Glu0.03, Glu0.06, Glu0.12, and Glu0.24, respectively. The control group, labeled as CK, consisted of 2 g of gibbsite in phosphate buffer without any Maillard precursors. The error bars in the scatter plots represent the standard deviation for each data point. The same applies to Figure 3 and Figure 4 below.

A higher *E*_4_/*E*_6_ ratio indicates a lower degree of aromatic condensation and humification [16]. Figure 1 depicts the variations in the *E*_4_*/E*_6_ ratio within the supernatant fluid during incubation with the addition of different concentrations of exogenous glucose. In the Gly0 treatment, the *E*_4_*/E*_6_ ratio significantly increased from 1.58 to 2.29 within the 0~18 h incubation period, followed by a gradual decline to 1.79 at the end of incubation. In contrast, the *E*_4_*/E*_6_ ratio in the Glu0.03 treatment decreased notably from 3.89 to 1.87 during the initial 0~6 h incubation phase, showing fluctuations between 1.89 and 2.06 with no clear trend, and ultimately settling at 1.94. Similarly, in the Glu0.06 treatment, the *E*_4_*/E*_6_ ratio decreased from 4.15 to 1.92 during the 0~18 h incubation interval, with a gradual increase from 28 h onwards, peaking at 240 h, and then decreasing to 1.82 until the end of incubation (360 h). The *E*_4_*/E*_6_ ratio in the supernatant fluid treated with Glu0.12 decreased from 3.50 to 2.04 during the initial 0~18 h period, displaying erratic fluctuations without a consistent pattern, and ultimately reaching 2.03. Similarly, the *E*_4_*/E*_6_ ratio in the Gly0.24 treatment experienced a sharp decrease from 5.25 to 2.42 during the 0~28 h time frame, followed by irregular fluctuations and ultimately dropping to 2.32. These *E*_4_*/E*_6_ ratios were much higher than those of artificial humic acids (HAs) from the abiotic humification of lignin-rich biogas, where the *E*_4_*/E*_6_ ratio was only 0.43 [17]. Although the *E*_4_*/E*_6_ ratios of the supernatant fluid decreased after the polyphenol–Maillard reaction and the structural condensation of HLA was enhanced, the degree of humification was lower than that of HAs from the abiotic humification of lignin-rich biogas. The *E*_4_*/E*_6_ ratio of the CK control group initially increased and then declined. By the end of incubation, the *E*_4_*/E*_6_ ratios of the Glu0 treatment and CK control group increased by 12.8% and 49.2%, respectively, compared to their initial values, indicating a weaker degree of aromatic condensation in the supernatant fluid. Conversely, the *E*_4_*/E*_6_ ratios of the Glu0.03, Glu0.06, Glu0.12, and Glu0.24 treatments decreased by 50.0%, 56.0%, 42.1%, and 55.8%, respectively, suggesting that the addition of glucose at concentrations of 0.03, 0.06, 0.12, and 0.24 mol/L could increase the degree of aromatic condensation in the supernatant fluid.

**Figure 2:**
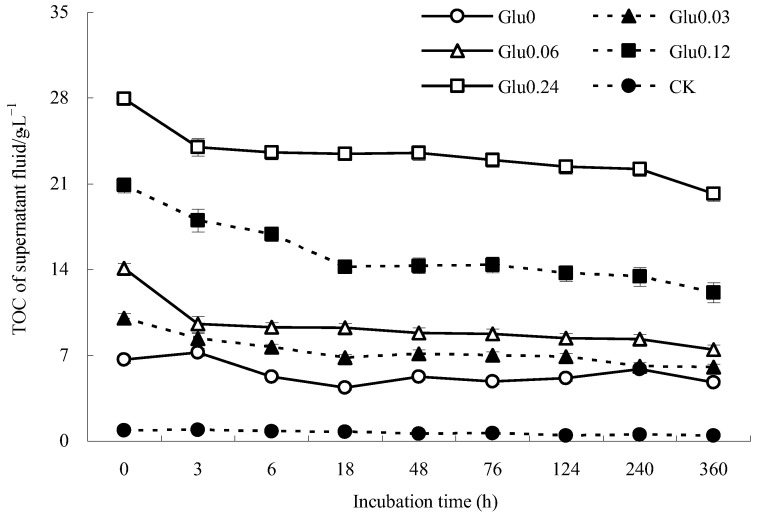
Impact of glucose concentration variations on the TOC content of the supernatant fluid.

Based on the data presented in Figure 2, the TOC content in the supernatant fluid of the Glu0 treatment increased from 6.6 to 7.2 g/L in the first 3 h and then fluctuated before reaching 4.8 g/L after 360 h of incubation. In contrast, the TOC content in the Glu0.03 treatment decreased significantly from 10.0 to 6.8 g/L in the initial 18 h and further dropped to 6.0 g/L after 360 h. The TOC content in the supernatant fluid of the Glu0.06 treatment gradually declined from 14.1 to 7.4 g/L throughout the incubation period. Similarly, the TOC content in the supernatant fluid of the Glu0.12 treatment decreased from 20.9 to 14.2 g/L in the first 18 h, remained relatively stable between 48 to 76 h, and then gradually decreased after 124 h, ultimately reaching 12.1 g/L. The TOC content in the Glu0.24 treatment steadily decreased from 28.0 to 20.2 g/L over the entire incubation period. In contrast, the TOC content in the supernatant fluid of the CK control showed minor fluctuations, ranging between 0.4 and 0.9 g/L. When comparing the TOC levels at the beginning and end of the 360 h incubation period, the Glu0, Glu0.03, Glu0.06, Glu0.12, Glu0.24, and CK control witnessed reductions of 28.0%, 39.9%, 47.2%, 42.1%, 27.8%, and 49.8% in TOC content, respectively, with the Glu0.24 treatment displaying the lowest decrease. When glucose concentrations were kept constant, the TOC content in the supernatant fluid decreased to a greater extent than that of *δ*-MnO_2_ as a catalyst. This loss of TOC content could be attributed to the superior adsorption capacity of gibbsite towards precursors compared to *δ*-MnO_2_ [18]. The addition of Maillard precursors effectively reduced the decline in TOC content in the supernatant fluid, resulting in lower TOC reductions compared to the CK control.

**Figure 3:**
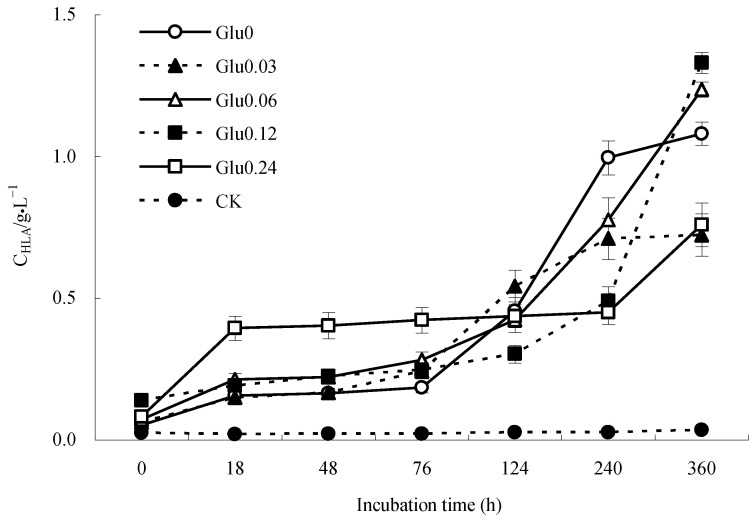
Impact of glucose concentration variations on C_HLA_ obtained from polyphenol–Maillard reaction.

**Figure 4:**
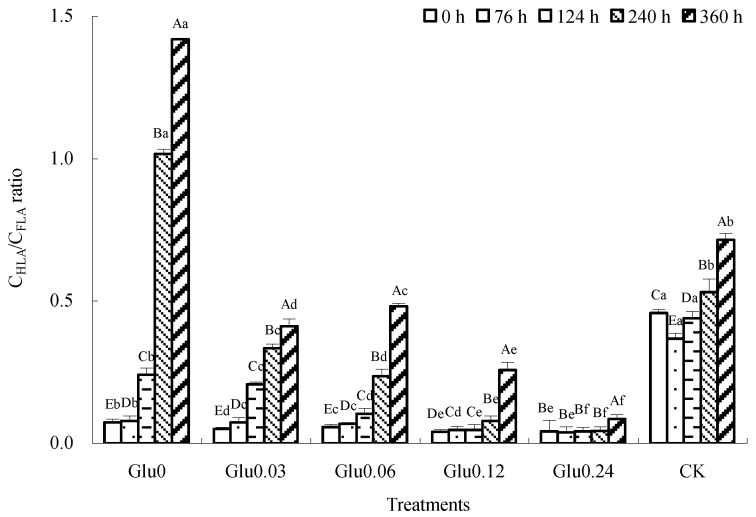
Impact of glucose concentration variations on the C_HLA_/C_FLA_ ratio obtained from polyphenol–Maillard reaction. Note: Capital letters represent significant differences (*p* < 0.05) in incubation period within the same treatment. Lowercase letters represent significant differences (*p* < 0.05) among treatments at the same time point.

### 2.2. C Content of Humic-like Acid (C_HLA_), Ratio of C Contents of Humic Acid and Fulvic Acid (C_HLA_/C_FLA_ Ratio) and FTIR Spectra of the HLA Obtained from Polyphenol–Maillard Reaction

Figure 3 illustrates the incubation progression, showing a relatively smooth fluctuation in the C_HLA_ of the CK control within the range of 0.02 to 0.03 g/L. After the introduction of Maillard precursors, each treatment exhibited a noticeable increase in C_HLA_ in response to varying glucose concentrations. At the end of the incubation period (360 h), the C_HLA_ for Glu0, Glu0.03, Glu0.06, Glu0.12, and Glu0.24 treatments, as well as the CK control, had increased by 2006.3%, 1062.7%, 1620.0%, 852.0%, 821.6%, and 40.8%, respectively, compared to their initial levels at 0 h. The presence of Maillard precursors in the treatments resulted in higher C_HLA_ than those observed in the CK control. Among the treatments, Glu0, which exclusively featured glycine and catechol, exhibited the most significant spike in C_HLA_, followed by the Glu0.06 treatment.

The C_HLA_/C_FLA_ ratio is used to assess the degree of HLS polymerization. An increase in the C_HLA_/C_FLA_ ratio implies that FLA is being transformed into HLA with a higher aromatic C structure and degree of condensation, which suggests a higher degree of HLS humification [19]. Figure 4 shows that the C_HLA_/C_FLA_ ratios for the different treatments followed distinct patterns. Specifically, the C_HLA_/C_FLA_ ratios for the Glu0, Glu0.03, Glu0.06, Glu0.12, and Glu0.24 treatments gradually increased, in contrast to the initial decrease followed by an increase in the CK control. After 360 h of incubation, the C_HLA_/C_FLA_ ratios for the Glu0, Glu0.03, Glu0.06, Glu0.12, and Glu0.24 treatments and the CK control had increased by 1833.4%, 720.3%, 756.1%, 548.3%, 101.9%, and 56.0%, respectively. Among these treatments, the Glu0 treatment showed the most significant increase in the C_HLA_/C_FLA_ ratio, followed by Glu0.06. The addition of Maillard precursors led to a greater increase in the C_HLA_/C_FLA_ ratio compared to the CK control, thereby enhancing the humification and condensation levels of the HLSs.

**Figure 5:**
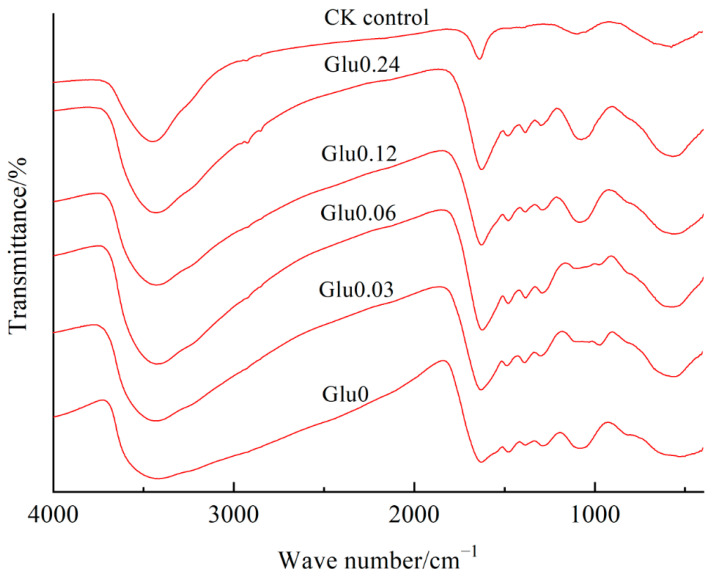
Impact of glucose concentration variations on FTIR spectra of HLA obtained from polyphenol–Maillard reaction.

Figure 5 illustrates that the prominent peak observed at 3431~3438 cm^−1^ signified the stretching vibration of the –OH orbital in gibbsite or the –OH group in interlayer water molecules, as well as the stretching vibration of hydroxyl group (–OH) in alcohols or phenolic compounds in HLA. The band observed at 1626~1635 cm^−1^ indicated the bending vibration of surface-adsorbed H_2_O in gibbsite or the stretching vibration of the aromatic ring skeleton C=O. A faint band at around 1481~1483 cm^−1^ was associated with the C–O asymmetric stretching of carboxyl groups, as noted by Song et al. [20]. The absorption peak at 1385~1387 cm^−1^ was linked to phenolic OH and the C=O stretching of carboxylates, while the band at 1288~1302 cm^−1^ corresponded to the C–O stretching of phenols and the C–O, O–H bending of carboxylic groups [21]. The peak located at 1090~1117 cm^−1^ indicated the stretching vibration of O–H, a feature exclusively observed in the CK control. Furthermore, the distinct absorption peak detected at 611~670 cm^−1^ was attributed to the lattice vibration of the layered Al–O bond in the HLA.

As summarized in Table 1, the absorption intensity of the peak at 3431~3438 cm^−1^ in HLA increased to different extents when Maillard precursors were exogenously added, compared to the CK control. This increase was attributed to the presence of stretching vibrations from the –OH groups in gibbsite or interlayer water molecules, as well as from the hydroxyl –OH groups in alcohol or phenolic compounds in the HLA. The combined effect of these vibrations resulted in a higher intensity of the peak at 3431~3438 cm^−1^ compared to the CK control. In comparison to the CK control group, the HLA sample treated with exogenous precursors exhibited an increased peak intensity at 1626~1635 cm^−1^ and a decreased intensity at 1385~1387 cm^−1^. This suggested a higher proportion of aromatic C structures and a lower amount of carboxyl groups. The addition of glucose, as well as its increasing concentration, led to a gradual increase in the vibration intensity of the absorption peak at 1090~1117 cm^−1^, representing polysaccharides. Additionally, there was an elevated vibration frequency of the absorption peak at 611~670 cm^−1^ due to the formation of Al–O bonds. Consequently, the absorption peaks at 1481~1483 cm^−1^ and 1288~1302 cm^−1^ showed varying degrees of decrease with the addition of glucose in each treatment. This indicated that the polyphenol–Maillard reaction resulted in varying degrees of reduction in the levels of carboxyl and phenolic groups in the HLA.

### 2.3. E_4_/E_6_ Ratio and Elemental Composition of the HLA Obtained from Polyphenol-Maillard Reaction

**Figure 6:**
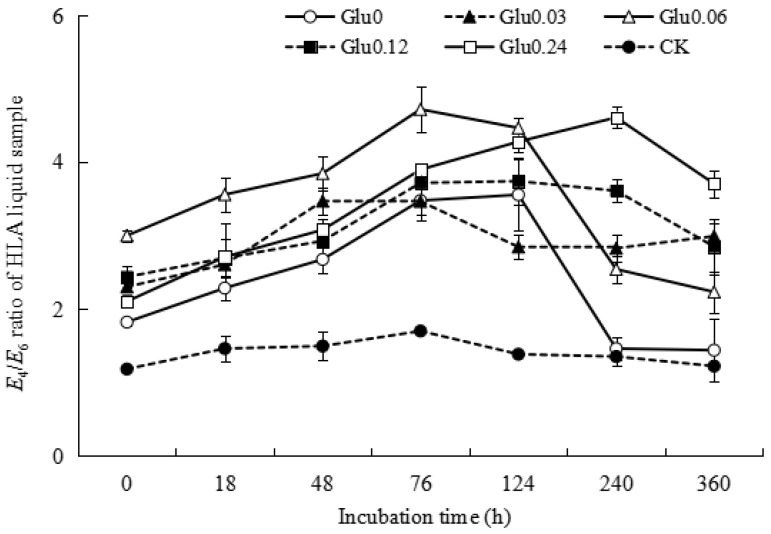
Impact of glucose concentration variations on the *E*_4_*/E*_6_ ratio of the HLA obtained from polyphenol–Maillard reaction. Note: Different glucose concentrations were denoted as Glu0, Glu0.03, Glu0.06, Glu0.12, and Glu0.24, representing 0, 0.03, 0.06, 0.12, and 0.24 mol/L concentrations, respectively. The control group, CK, consisted of 2 g of gibbsite in a phosphate buffer without any Maillard precursor. The error bars on the scatter plots indicate the standard deviation for each data point.

Figure 6 illustrates the trends in *E*_4_*/E*_6_ ratios for HLA derived from different treatments and CK control. They demonstrated an initial increase followed by a decrease during the incubation period. In the Glu0 treatment, the *E*_4_/*E*_6_ ratio for HLA increased from 1.82 to 3.56 within the first 124 h and then decreased to 1.44 by the end of the incubation. For the Glu0.03 treatment, the *E*_4_/*E*_6_ ratio increased from 2.30 to 3.47 over the initial 76 h, remained stable with some fluctuations, and eventually reached 2.99 at the completion of incubation. In the Glu0.06 treatment, the *E*_4_/*E*_6_ ratio for HLA increased from 3.00 to 4.72 within the first 76 h, gradually declining to 2.23 by the end. In the Glu0.12 treatment, the *E*_4_/*E*_6_ ratio increased from 2.44 to 3.74 in the first 124 h, before stabilizing at 2.85. Under the Glu0.24 treatment, the *E*_4_/*E*_6_ ratio rose from 2.10 to 4.61 over 240 h, eventually settling at 3.71. In the CK control group, the *E*_4_/*E*_6_ ratio increased from 1.18 to 1.70 in the first 76 h and then decreased to 1.22 by the end of the incubation period. At the end of the 360 h incubation, compared to the initial readings, the *E*_4_/*E*_6_ ratios for HLA from Glu0 and Glu0.06 decreased by 21.0% and 25.6%, respectively, while those from the Glu0.03, Glu0.12, and Glu0.24 treatments and the CK control increased by 30.0%, 16.9%, 76.2%, and 3.4%, respectively. These findings suggested that the absence of glucose or glucose at a concentration of 0.06 mol/L was most effective in promoting the aromatic condensation of HLA molecules. Conversely, glucose concentrations of 0.03, 0.12, and 0.24 mol/L were found to decrease the aromatic condensation of HLA molecules.

Table 2 shows that the CK control group was treated with the addition of gibbsite and no other precursor in phosphate buffer. As a result, the extraction of HLA was impeded, resulting in a lack of corresponding elemental information. The Glu0 treatment was used as the reference point.

Compared to the Glu0 and Glu0.06 treatments, the HLA extracted from the remaining incubation medium of the Glu0.03, Glu0.12, and Glu0.24 treatments exhibited higher H/C ratios and lower O/C ratios. This indicated that the HLA molecules treated with Glu0 and Glu0.06 had increased aromaticity and decreased polarity, consistent with findings from previous studies [22,23]. The presence of glucose in the treatments resulted in an increase in N-containing compounds within the HLA molecules, leading to a lower C/N ratio compared to the Glu0 treatment. Additionally, higher concentrations of glucose were found to stimulate the production of more N-containing compounds in the HLA molecules.

## 3. Materials and Methods

### 3.1. Materials and Reagents

Glucose (C_6_H_12_O_6_), glycine (C_2_H_5_NO_2_), and catechol (C_6_H_6_O_2_) were used as polyphenol–Maillard precursors in this study. These precursors were obtained from Sinopharm Chemical Reagent Co., Ltd., located in Shanghai, China. Catechol was chosen as the representative polyphenol for our experimental setups due to its frequent occurrence of 1,2-dihydroxy-substituted phenolic compounds in nature. It is a byproduct of lignin decomposition reactions and can be found in various natural sources such as plant canopies, root leachates, and microbial metabolites [24]. Glycine was selected as the model amino acid compound for this study because of its abundance in soils and its significant role as a component of microbial cell walls [24]. Glucose was opted for as it is a primary decomposition product resulting from the natural breakdown of cellulose, which is a highly abundant substrate in terrestrial environments [6].

Gibbsite, which is represented by the chemical formula Al(OH)_3_, was synthesized using the AlCl_3_-NaOH method [25].

For the preparation of a 0.2 mol/L phosphate-buffered solution (pH 8.0), a total of 5.3 mL of 0.2 mol/L NaH_2_PO_4_ was mixed carefully with 94.7 mL of 0.2 mol/L Na_2_HPO_4_. Additionally, 0.02% thimerosal (C_9_H_9_HgNaO_2_S) was included in the mixture.

### 3.2. Experimental Design

**Figure 7:**
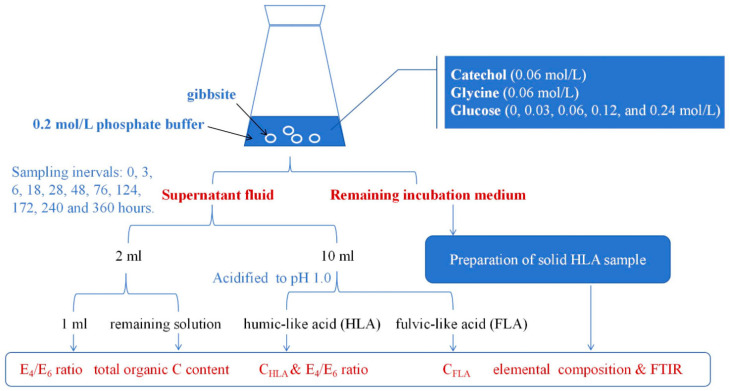
Schematic diagram of experimental design.

The experimental design is shown in Figure 7. Strict aseptic conditions were maintained throughout the experiments to ensure the predominance of abiotic transformation. Before usage, all glassware, phosphate buffer, and other equipment were sterilized through autoclaving. Then, 500 mL conical flasks were prepared for the experiment. Each flask was filled with 250 mL of 0.2 mol/L phosphate buffer, and 2 g of gibbsite was added to each solution. Catechol and glycine were then added at a concentration of 0.06 mol/L and subjected to incubation with varying levels of glucose (0, 0.03, 0.06, 0.12, and 0.24 mol/L). These treatments, involving different glucose concentrations, were labeled as Glu0, Glu0.03, Glu0.06, Glu0.12, and Glu0.24. The control group (CK) was established by adding 2 g of gibbsite solely to the phosphate buffer. Each reaction was performed in triplicate to ensure accuracy.

The liquid shake-flask incubation was performed in a sterile environment, with a rotation speed of 150 rpm. Throughout the incubation process, a 2 mL portion of the supernatant fluid was taken from each system at specified time intervals (0, 3, 6, 18, 28, 48, 76, 124, 172, 240, and 360 h). The extracted samples were then subjected to high-speed centrifugation at 16,000 rpm for 5 min. Subsequently, 1 mL of the sample was diluted to 25 mL and the absorbances at 465 and 665 nm (*E*_4_ and *E*_6_) were measured using a UV–visible spectrophotometer (TU-1900, Beijing Purkinje General Instrument Co., Ltd., Beijing, China). The *E*_4_/*E*_6_ ratio was calculated, which serves as an indicator of optical properties and provides insight into the degree of aromatic condensation and humification in a liquid sample [16]. The remaining solution was used to determine the total organic C (TOC) content using a Vario TOC cube from Elementar in Germany. Furthermore, another 10 mL portion of the supernatant fluid was collected at the aforementioned time intervals and acidified with 1.0 mol/L HCl to reach a pH of 1.0. The acidified solution was allowed to equilibrate for 24 h, followed by centrifugation. The resulting supernatant was identified as fulvic-like acid (FLA) and was neutralized before dilution. The precipitate was identified as humic-like acid (HLA), which was dissolved in a 0.1 mol/L NaOH solution, neutralized, and diluted to create an HLA liquid sample. The *E*_4_/*E*_6_ ratio of the HLA liquid sample was determined using the same method as the supernatant fluid. The C contents of HLA and FLA (C_HLA_ and C_FLA_) were determined using a TOC analyzer, and the C_HLA_/C_FLA_ ratio was calculated. After the 360 h incubation period, the HLA was isolated from the remaining incubation medium in the conical flasks by acidification at a pH of 1.0. The HLA was then dissolved in a 0.1 mol/L NaOH solution and treated with a mixture of HCl (6%, *v*/*v*) and HF (6%, *v*/*v*) to remove inorganic impurities. Subsequently, the solution was centrifuged at 16,000 rpm for 10 min. The solid HLA samples were purified using electrodialysis and freeze-drying processes, and then further processed by grinding and sieving to achieve a particle size of 0.01 mm for subsequent analysis.

### 3.3. Analytical Methods and Data Analyses

The elemental composition of a solid HLA sample, specifically the percentages of C, H, O, and N, was determined using an elemental analyzer (PE 2400II CHNS/O, Perkin-Elmer, Waltham, MA, USA). The structural characterization of the solid HLA samples was conducted using FTIR spectrophotometry (FTIR-850, Tianjin Gangdong Sci & Tech Development Co., Ltd., Tianjin, China). FTIR spectra were acquired in the range of 400 to 4000 cm^−1^ and analyzed using FTIR 850 software. The results were presented using Origin 2021 software and Microsoft Excel 2003 for graphical representation. Statistical analysis of the data was performed using SPSS 18.0 (ANOVA) with one-way analysis of variance and the least significant difference (LSD) test.

## 4. Discussion

The introduction of glucose during the polyphenol–Maillard reaction increased the degree of aromatic condensation in the supernatant fluid. The order of impact was as follows: Glu0.06 > Glu0.24 > Glu0.03 > Glu0.12. When heated, glycine and glucose reacted to form Schiff bases through amino acid–carbonyl condensation [26]. Subsequently, they underwent a series of reactions and polymerized with amine compounds to form HLSs. In contrast, the degree of aromatic condensation in the supernatant fluid from Glu0 and CK control was lower. With the presence of sugars, smaller molecules like phenols and amino acids underwent further reactions, polymerization, and partial condensation facilitated by gibbsite, leading to a higher degree of humification. Supramolecular bonds were formed among humic acid molecules of different sizes, resulting in the formation of a humic network [27]. According to Hardie et al. [6], increasing the molar ratio of glucose to catechol and glycine in the combined catechol–Maillard system enhanced the production of low-molecular-weight, highly aliphatic carboxylic products in the supernatant fluid. However, under the experimental conditions, the addition of glucose in the presence of catechol and glycine, with the help of gibbsite catalysis, increased the condensation degree of aromatic C in the supernatant fluid. This could be attributed to the aggregation of the precursors caused by gibbsite adsorption or the participation of its own functional groups in the polyphenol–Maillard reaction. While the TOC content in the supernatant fluid of each treatment decreased to varying degrees after incubation due to the adsorption of Maillard precursors by gibbsite, the addition of precursors partially hindered the loss of TOC content in the supernatant fluid. Furthermore, the supplementation of C-containing precursors, in conjunction with the formation of Al–C particles primarily facilitated by the interaction between gibbsite and precursors, demonstrated the ability to impede the loss of TOC content in the supernatant fluid, thereby enhancing C sequestration [28]. Qi et al. [12] observed that incorporating catechol into chicken manure composting resulted in a reduction in organic matter loss and an increase in recalcitrant component levels.

Following the incubation process, it was observed that the C_HLA_ and C_HLA_/C_FLA_ ratio increased to varying degrees compared to the CK control group. However, the introduction of Maillard precursors resulted in a more significant enhancement of C_HLA_ and the C_HLA_/C_FLA_ ratio. This implied that FLA was transformed into HLA with a higher aromatic C structure and degree of condensation, ultimately leading to an enhancement in the overall humification degree of HLSs. Notably, the Glu0 treatment, consisting only of glycine and catechol, showed the most significant effect, followed by the Glu0.06 treatment. The primary components of biomass waste, including sugars, amino acids, and phenols, were frequently utilized as representative humic precursors in abiotic humification models to generate HLA. These exogenous precursors could participate in humification pathways, such as the polyphenol–Maillard reaction, thereby directly facilitating the production of HSs and HA, as outlined by Chen et al. [29]. Zhang et al. [30] observed that the addition of catechol to the Maillard system could accelerate the generation of darkening substances. However, the current research revealed that a higher amount of C_HLA_ was generated when only catechol and glycine (Glu0 treatment) were present, with catechol playing a primary role in the formation of HLA. Zou et al. [31] investigated the specific functions of MnO_2_ and O_2_ in the formation of HSs through the oxidative polymerization of catechol and glycine in the absence of glucose. They found that significant amounts of FA and HA were formed only in the presence of O_2_, whereas the combination of O_2_ and MnO_2_ notably boosted HA production. This discovery confirmed that polyphenols served as fundamental components in HLA formation [32]. Polyphenols had the ability to undergo oxidation, resulting in the formation of quinones which then reacted with aromatic amino acids to produce HSs or polymerize with polysaccharides to create covalent compounds that played a role in HS production [33]. Additionally, naturally occurring metal oxides like gibbsite often came into contact with O_2_, leading to a synergistic effect that enhanced the oxidative polycondensation of polyphenols and subsequently boosted the production of HA [34]. The polyphenol–Maillard reaction was the main contributor to HA polymerization [32]. The presence of gibbsite (Al^3+^), known for its Lewis acid properties, served to enhance the yield of HLA [17]. The increase in the C_HLA_/C_FLA_ ratio more accurately indicated the shift from FLA to HLA, which exhibited a higher degree of condensation, thereby enhancing the quality of the HLSs [9]. This rise in the C_HLA_/C_FLA_ ratio more explicitly reflected the transition from unstable HS component molecules (i.e., FA) to more mature HS components (i.e., HA) [35], subsequently enhancing the humification of the HLSs. The incorporation of glycine and catechol, without or with the addition of glucose at a concentration of 0.06 mol/L, was shown to be more favorable for enhancing the degree of condensation of HLA molecules or reducing the level of oxidation. On the other hand, the other three glucose concentrations (0.03, 0.12, and 0.24 mol/L) were more favorable for the decomposition of HLA molecules. An elevated concentration of glucose, when combined with glycine and catechol, could trigger the production of additional N-containing compounds in the HLA molecules. A greater quantity of aromatic and less polar compounds was generated from the HLA molecules in the Glu0.03, Glu0.12, and Glu0.24 treatments compared to those in the Glu0 and Glu0.06 treatments. These findings highlighted the involvement of certain intermediary compounds (e.g., amino acids and carbohydrates) in the depolymerization and oxidation processes, resulting in an increase in amidogen and hydroxyl groups bonded to aromatic rings [36]. This characteristic feature indicated the polyphenol–Maillard reaction, which encompassed the breakdown, reorganization, and bonding of proteins and sugars to form N-containing heterocycles [4].

The participation of Maillard precursors strengthened the stretching vibration of the hydroxyl group (–OH). The addition of glycine and catechol, along with different concentrations of glucose (0, 0.03, 0.06, 0.12, or 0.24 mol/L), increased the aromatic C structure in the HLA via the polyphenol–Maillard reaction, while reducing the number of carboxyl groups. Carboxyl groups, crucial intermediate products in the formation of HSs, played a significant role in increasing the quantity of aliphatic compounds and the degree of unsaturation in HSs [9]. The lower proportion of carboxyl groups further confirmed their contribution to the development of HSs. This difference might be attributed to the release of Al ions by gibbsite, which occurred due to the generation of electrons during the oxidation of humic precursors. The release of Al ions facilitated the formation of •OH, which enhanced the conversion of humus precursors [37]. •OH not only had superior oxidation capacity in promoting the opening of aromatic rings and the oxidative polycondensation of phenolic and amino acids but also positively influenced the oxidative polymerization of phenols [38]. With the introduction of Maillard precursors, an increase in glucose concentration led to the higher accumulation of polysaccharides in the HLA and the formation of Al–O bonds, consistent with the findings of Zhang et al. [35]. The presence of glucose was found to enhance the signal intensity of O-alkyl and nonpolar alkyl groups in soil organic matter spectra, indicating increased glucose accumulation. Polysaccharide hydrolysis products, known as reducing sugars, played a crucial role in the synthesis of polyphenols and the condensation of HSs [39]. However, the levels of phenolic groups and phenols in the HLA showed varying degrees of decrease. Polyphenols, labile precursors for HS formation, exhibited higher reactivity compared to reducing sugars and amino acids under abiotic conditions [40]. Studies have indicated that decreased amounts of polyphenols promote HS formation, and a significant negative relationship was found between polyphenol concentration and humification indices in all cases [41].

Gibbsite has the ability to expedite the oxidation of humus precursors, accept electrons from organic C, and function as binding sites for the association of organic C, leading to the formation of mineral-associated organic C with enhanced stability [14,42]. In this process, a cation bridge composed of Al–O was established to provide protection against chemical or biological degradation. The catalytic influence of Fe and Mn (oxyhydr) oxides could be attributed to an adsorptive mechanism that favorably clustered and oriented the reactants at mineral surfaces, thereby increasing the reaction rate for a redox reaction involving glucose and the minerals that produced dissolved Fe(II) and Mn(II) for bridging effects [43]. The oxidized glucose reacted with glycine to form a Schiff base, which served as a precursor to polyphenol–Maillard reaction byproducts. The HLA molecules under the Glu0 and Glu0.06 treatments showed increased condensation and decreased oxidation levels. The addition of higher concentrations of glucose could prompt the formation of more N-containing compounds in the HLA. This variation might have been due to the alteration of ammonia nitrogen during the humification process [44]. Ultimately, these products were converted into aromatic N-containing compounds resembling HSs [23].

## 5. Conclusions

The introduction of glucose in the polyphenol–Maillard reaction increased the degree of aromatic condensation of the supernatant fluid. The hierarchy of effects was as follows: Glu0.06 > Glu0.24 > Glu0.03 > Glu0.12. The addition of Maillard precursors mitigated the declined trend in TOC contents in the supernatant fluid caused by the adsorption of gibbsite. This resulted in a more significant increase in C_HLA_ level and the C_HLA_/C_FLA_ ratio, ultimately enhancing the humification degree of the HLSs. When glycine and catechol co-existed, the polyphenol–Maillard reaction enhanced the degree of molecular condensation of HLA without the addition of glucose or with a glucose concentration of 0.06 mol/L. The addition of glycine and catechol with a higher glucose concentration led to the formation of more N-containing compounds within HLA. The presence of Maillard precursors increased the stretching vibration of the hydroxyl group –OH in HLA molecules. It also raised levels of aromatic C structures, facilitated the accumulation of polysaccharides, and encouraged the formation of Al–O bonds in HLA. However, the phenol and carboxyl groups in HLA molecules were reduced to some extent.

## Figures and Tables

**Table 1:** FTIR relative intensities (% of total area) for the HLA obtained from polyphenol–Maillard reaction as influenced by varying concentrations of glucose.

	Wavenumbers (cm^−1^)	3431~3438	1626~1635	1481~1483	1385~1387	1288~1302	1090~1117	611~670
Treatments								
Glu0		70.9	16.0	1.46	0.55	2.29	1.63	3.59
Glu0.03		77.8	15.3	0.70	0.59	1.84	3.45	4.95
Glu0.06		75.8	15.9	0.36	0.52	0.85	5.45	6.55
Glu0.12		71.7	14.8	1.16	0.55	1.42	6.52	7.83
Glu0.24		75.4	14.2	0.24	0.41	0.50	6.83	8.31
CK control		70.7	14.0	1.59	0.69	2.31	1.52	3.21

**Table 2:** Elemental composition of the HLA obtained from the polyphenol–Maillard reaction under varying concentrations of glucose.

Treatments	H/C Ratio	C/N Ratio	O/C Ratio
Glu0	1.39 ± 0.03 d	19.3 ± 0.8 a	1.06 ± 0.03 a
Glu0.03	1.45 ± 0.05 c	18.7 ± 0.3 b	1.01 ± 0.02 b
Glu0.06	1.38 ± 0.02 d	17.6 ± 0.6 c	1.07 ± 0.05 a
Glu0.12	1.52 ± 0.04 b	15.9 ± 0.2 d	0.98 ± 0.05 c
Glu0.24	1.61 ± 0.06 a	14.3 ± 0.4 e	0.94 ± 0.04 d

Note: The data in the table are reported as mean ± standard deviation. Lowercase letters are used to indicate statistically significant differences among the treatments (*p* < 0.05).

## Data Availability

Data are contained within the article.
